# Stepwise Induction of Inner Ear Hair Cells From Mouse Embryonic Fibroblasts via Mesenchymal- to-Epithelial Transition and Formation of Otic Epithelial Cells

**DOI:** 10.3389/fcell.2021.672406

**Published:** 2021-06-17

**Authors:** Qiong Yang, Haosong Shi, Yizhou Quan, Qianqian Chen, Wang Li, Li Wang, Yonghui Wang, Zhongzhong Ji, Shan-Kai Yin, Hai-Bo Shi, Huiming Xu, Wei-Qiang Gao

**Affiliations:** ^1^State Key Laboratory of Oncogenes and Related Genes, Renji-Med X Stem Cell Research Center, Ren Ji Hospital, School of Medicine, Shanghai Jiao Tong University, Shanghai, China; ^2^Department of Otorhinolaryngology, The Sixth People’s Hospital of Shanghai, Shanghai Jiao Tong University, Shanghai, China; ^3^School of Biomedical Engineering and Med-X Research Institute, Shanghai Jiao Tong University, Shanghai, China

**Keywords:** hair cell regeneration, mesenchymal-to-epithelial transition (MET), hearing loss, mouse embryonic fibroblasts, transdifferentiation

## Abstract

Although embryonic stem cells or induced pluripotent stem cells are able to differentiate into inner ear hair cells (HCs), they have drawbacks limiting their clinical application, including a potential risk of tumourigenicity. Direct reprogramming of fibroblasts to inner ear HCs could offer an alternative solution to this problem. Here, we present a stepwise guidance protocol to induce mouse embryonic fibroblasts to differentiate into inner ear HC-like cells (HCLs) via mesenchymal-to-epithelial transition and then acquisition of otic sensory epithelial cell traits by overexpression of three key transcription factors. These induced HCLs express multiple HC-specific proteins, display protrusions reminiscent of ciliary bundle structures, respond to voltage stimulation, form functional mechanotransduction channels, and exhibit a transcriptional profile of HC signature. Together, our work provides a new method to produce functional HCLs *in vitro*, which may have important implications for studies of HC development, drug discovery, and cell replacement therapy for hearing loss.

## Introduction

Inner ear hair cells (HCs), the mechanoreceptors for perception of sound and balance, can convert the sound or motion into electrochemical signals (Carey and Amin, 2006; Costa et al., 2015). They are terminally differentiated cells and are positioned in the sensory epithelium of the inner ear. The latter grows from the otic placode during embryogenesis (Zhai et al., 2005; Kelley, 2006). Unfortunately, HCs are easily injured by noise, ototoxins, genetic predisposition, and aging and hardly have a capability to regrow in adult mammals (Schacht, 1986; Dror and Avraham, 2009; Smith et al., 2016). As a result, millions of people worldwide are permanently debilitated by hearing loss and balance problems (Ma et al., 2019). Therefore, development of a novel method of producing new HCs *in vitro* may provide a cell replacement therapy method and serve as a helpful system for our studies of HC biology, disease, and regeneration.

By far, stem cells are believed to be a promising source for cell replacement therapy to treat HC loss (Géléoc and Holt, 2014). For HC regeneration, it has been shown that either embryonic stem cells (ESCs) or induced pluripotent stem cells (iPSCs) could be induced to differentiate into inner ear HCLs (Li et al., 2003; Oshima et al., 2010; Koehler et al., 2017; Ouji et al., 2017). However, clinical application of ESCs is troubled by its possible immune rejection and moral and safety worries. Similarly, iPSCs are also limited in clinical use due to its time-consuming and genetic instability that lead to tumor formation (Lanza, 2007; Knoepfler, 2009). In addition to ESCs and iPSCs, some recent studies showed that HCLs could be obtained from either the differentiation of stem cells in the inner ear or a direct transdifferentiation of supporting cells (SCs) in the inner ear (Zheng and Gao, 2000; White et al., 2006; Liu Q. et al., 2016), suggesting that inner ear stem cells and SCs might be considered sources for clinical research and trial application. However, the use of postnatal inner ear stem cells and SCs still encounters a great challenge attributable to the insufficiency of their supply and conceivable ethical concerns (Burns et al., 2012; Liu Q. et al., 2016). In order to overcome the concerns mentioned above, investigators have focused on seeking for additional appropriate cell reservoir as an alternative source. A number of recent investigations reported that fibroblasts have some distinctive advantages, such as less of ethical concern, convenience, accessibility, fast proliferation, and relative guarantee of safety. They are somatic cells and are capable of further converting to cell type of interest that can be used for future auto-transplantation cell therapy. For instance, overexpression of lineage-specific transcription factors (TFs) can directly convert fibroblasts into some other lineages, including neurons (Vierbuchen et al., 2010; Ambasudhan et al., 2011), hepatocytes (Sekiya and Suzuki, 2011; Huang et al., 2011), cardiomyocytes (Ieda et al., 2010; Fu et al., 2013), Sertoli cells (Buganim et al., 2012), and blood progenitors (Szabo et al., 2010). The converted cells can acquire relevant physiological functions. However, up to now, it remains unclear whether and how mouse fibroblasts can be successfully converted into functional HCs.

Previously, several TFs have been shown to act as determinants for HC fate and to influence HC differentiation during inner ear development (Schimmang, 2013). Inner ear HC differentiation requires Atoh1 (Zheng and Gao, 2000), as well as Sox2, Eya1, and Six1 (hereafter referred to as SES), and the latter three genes are co-expressed in sensory progenitors. Mutations in these four genes cause sensorineural hearing loss (Xu et al., 1999; Zheng and Gao, 2000; Ozaki et al., 2004; Dabdoub et al., 2008; Ahmed et al., 2012). Recent reports indicate that SES proteins interact directly and cooperatively to regulate the Atoh1 enhancer, leading to expression of Atoh1 and production of differentiated HCs. In detail, Sox2 and Six1 directly bind to the Atoh1 enhancer, while the transcriptional coactivator Eya1 establishes a bridge between Sox2 and Six1. Together, these findings confirm that SES work together with Atoh1 during HC fate determination in the inner ear (Ahmed et al., 2012; Schimmang, 2013).

In the present study, we applied a stepwise strategy to induce mouse embryonic fibroblasts (MEFs) into HCLs. Firstly, MEFs were obtained and induced initially to undergo mesenchymal-to-epithelial transition (MET) by a small-molecule transforming growth factor beta (TGF-β) inhibitor, SB431542. Subsequently, by using a combination of three TFs SES, the epithelial cells derived from the MEFs were induced to become a population of otic epithelial cells (OECs). These OECs were then capable of differentiating into mechanosensitive sensory HCLs. Importantly, via such sequential steps, the MEF-derived HCLs expressed HC-specific genes, displayed ciliary bundle structures, could be permeated rapidly by FM1-43, and exhibited a transcriptional profile of HC signature based on RNA sequencing analyses. Furthermore, these HCLs were responsive to voltage stimulation. Therefore, we provide in this study a novel stepwise method to successfully convert mouse somatic cells into functional HCLs.

## Results

### Preparation and Characterization of Mouse Embryonic Fibroblasts

**FIGURE 1:**
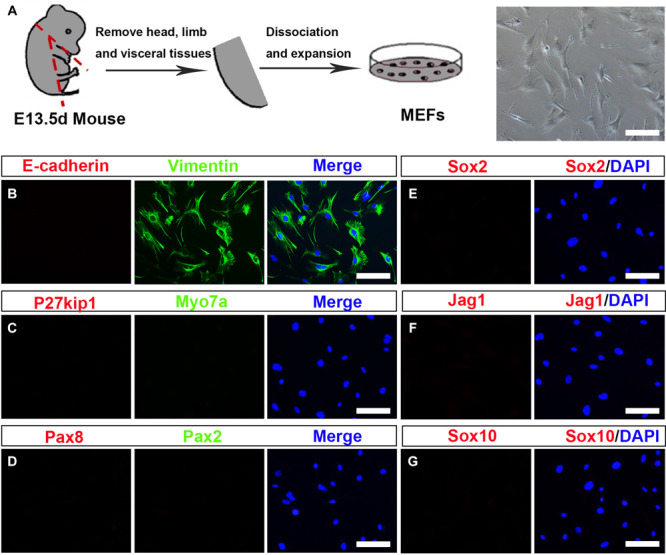
Establishment and characterization of mouse embryonic fibroblasts (MEFs). **(A)** Fibroblasts isolated from mouse embryos were dissociated and cultured under standard fibroblast culture conditions. The fibroblasts were passaged and frozen, and cells passaged at generation 2 or 3 were used for experiments. **(B–G)** Immunostaining of the MEF cells for Vimentin (green) and E-cadherin (red). Hair cells (HCs), supporting cell, and otic epithelial cell (OEC) markers included Myo7a (green), P27^kip^ (red), Pax2 (green), Pax8 (red), Sox2, Jag1, and Sox10 (scale bar, 25 μm).

To investigate whether MEFs can be used to convert into inner ear HCs, first, we prepared the primary MEFs from embryonic day 13.5 (E13.5) C57BL/6J mice (Vierbuchen et al., 2010). The head, the limb, the visceral tissues, and all red organs were removed, and the remaining tissue was dissociated and cultivated in Dulbecco’s modified Eagle’s medium (DMEM) in which we supplemented with 10% fetal bovine serum (FBS) and 1% penicillin (Figure 1A). MEFs were used for the subsequent conversion experiments at passage 2 or 3. To study if the MEFs that we isolated were pure fibroblasts without contamination of any HCs, SCs, or OECs, we characterized them in the cultures by immunofluorescence staining with Vimentin, E-cadherin, Myo7a, P27^kip^, Pax2, Pax8, Sox2, Jag1, and Sox10 antibodies. As shown in Figure 1B, the MEFs were E-cadherin negative and Vimentin positive. In addition, they were negative to Myo7a, P27^kip^, Pax2, Pax8, Sox2, Jag1, and Sox10 antibodies, markers of HCs, SCs, or OECs (Figures 1C–G). Thus, the MEFs that we prepared were pure fibroblasts and were not contaminated by HCs, SCs, and OECs.

### Transforming Growth Factor Beta Inhibitor Induces Mouse Embryonic Fibroblasts to Undergo a Mesenchymal-To-Epithelial Transition

**FIGURE 2:**
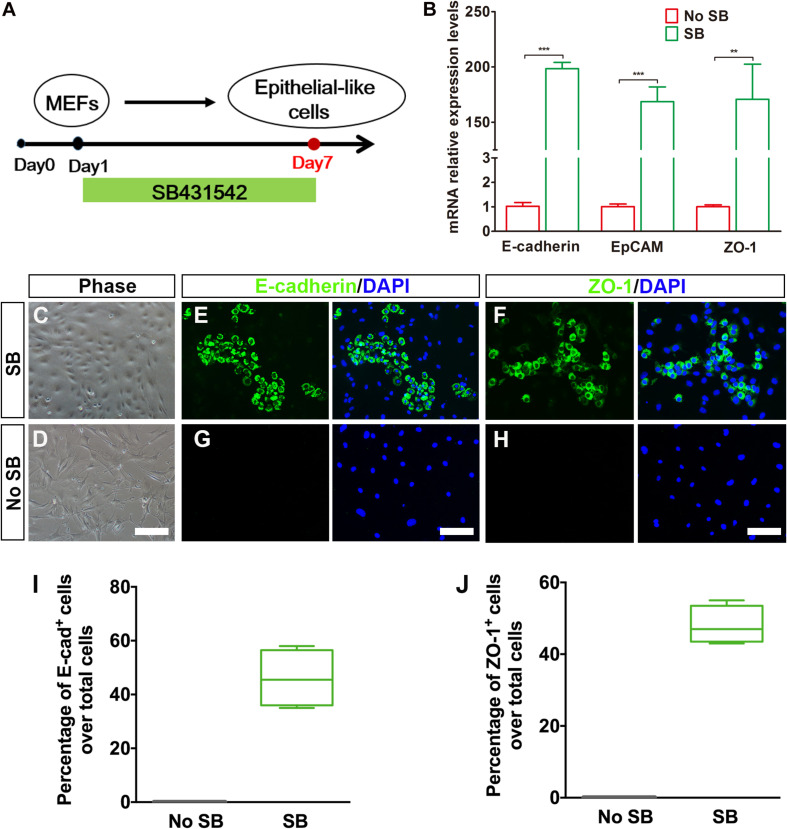
Generation of epithelial-like cells from mouse embryonic fibroblasts (MEFs) by a TGF-β inhibitor. **(A)** An induction protocol describing the SB431542 treatment timeline. **(B)** Quantitative real-time PCR analysis of mRNA levels of E-cadherin, EpCAM, and ZO-1 from MEF-derived epithelial-like cells. MEFs in normal cultures were used as control group, no SB. Data were collected from at least three separate experiments and are shown as means ± SEM. Statistical significance was tested by Student’s *t*-test and expressed as **p < 0.01, ***p < 0.001 compared with the control. **(C,D)** Phase-contrast images of epithelial-like cells (ELCs) and MEFs showing epithelial and mesenchymal cell morphologies, respectively. **(E,F)** Immunostaining analysis of E-cadherin and ZO-1 expression at day 7 in SB-treated cells. Strong upregulation of E-cadherin **(E)** and ZO-1 **(F)** was detected in SB-treated cells. **(G,H)** Immunostaining analysis of E-cadherin and ZO-1 expression at day 7 in no SB treatment cells. No such staining was observed in MEFs without SB treatment. **(I,J)** Frequencies of immunopositive cells for E-cadherin **(I)** and ZO-1 **(J)** in MEF-derived cells with or without SB. Without SB, immunopositivity for E-cadherin and ZO-1 was absent. In contrast, a remarkable number of immunopositive cells were detected in MEF-derived cells with SB. Error bars represent the SEM. *n* = 4 (scale bar, 25 μm).

Considering that fibroblasts and inner ear HCs belong to different cell lineages, we postulated that the conversion of MEFs into inner ear HCs might go through a stepwise manner with an initial MET step. In this regard, we designed our experiment by first inducing the MEFs to become epithelial cells. To achieve this goal, we decided to add a small molecule SB431542 (an inhibitor of TGF-β signaling) to the culture medium, which has been shown to facilitate MET of MEFs and to improve the nuclear reprogramming of mouse fibroblasts (Li et al., 2010). The experimental protocol is described in Figure 2A.

To assess the MET of MEFs, we dissociated MEFs and replated them onto poly-L-lysine-coated glass slips in DMEM-containing serum and then replaced with serum-free DMEM/F12 medium plus N2, B27 supplements, and SB431542 (SB) 1 day later. Our quantitative real-time PCR (qRT-PCR) analysis showed an upregulation of E-cadherin gene and a downregulation regulation of Vimentin, a marker for fibroblast, at 3 days post SB treatment in the cultures as compared with the untreated group (Supplementary Figure 1A). Such expression pattern became increased at 5 days post SB treatment (Supplementary Figure 1B). To determine whether there was a similar expression pattern for E-cadherin and Vimentin proteins, we performed double immunostaining at 5 days post SB treatment in the cultures. As shown in Supplementary Figure 1C, while a considerable number of the cells became E-cadherin positive, many of the cells remained Vimentin positive. In contrast, no E-cadherin-positive cells were seen in the control cultures. At this time point, the E-cadherin staining appeared to be punctate, implying an early stage of MET. To find out whether as time proceeds the MET becomes more complete, we did qRT-PCR analysis for E-cadherin as well as EpCAM and ZO-1, two additional epithelial markers. As shown in Figure 2B, the expression of all of the three epithelial marker genes was significantly upregulated as compared with the control cultures. Furthermore, the MEFs with SB treatment exhibited small, compact cell bodies with an epithelial-like morphology (Figures 2C,D). These cells also showed positive immunoreactivity to E-cadherin and ZO-1 antibodies (Figures 2E,F). In contrast, no such staining was observed in MEFs without SB treatment (Figures 2G,H). Moreover, the E-cadherin staining was no longer punctate but rather showed a continuous membrane pattern. Notably, widespread expression of E-cadherin and ZO-1 was only detected in MEFs treated with SB (46 ± 5.4% and 48 ± 2.6% over total cells, respectively) and never seen in MEFs without SB treatment (Figures 2I,J). Thus, the TGF-β signaling inhibitor facilitates MEFs to gradually undergo a MET so as to form epithelial-like cells.

### Conversion of Mouse Embryonic Fibroblast-Derived Epithelial-Like Cells to Otic Epithelial Cells by Transduction of Sox2, Eya1, and Six1

Recent experiments have demonstrated that overexpression of lineage-specific TFs was able to lead to cell-fate changes in various types of somatic cell. For example, Ahmed and colleagues found that a combination of Eya1 and Six1 induces an HC fate in Sox2-expressing non-sensory cells in mouse cochlear explants (Ahmed et al., 2012). In addition, SES are co-expressed in sensory progenitors. If these genes are mutated, an early arrest of otic development can happen in mice (Xu et al., 1999; Ozaki et al., 2004; Dabdoub et al., 2008; Schimmang, 2013). After considering the TFs that are crucial for HC development and regeneration, we decided to examine whether a combination of SES can program MEF-derived epithelial-like cells to become OECs. To establish a “tunable” SES overexpression system, we first generated doxycycline (Dox)-inducible lentiviruses expressing SES (Supplementary Figure 2A). Then we transduced the cultured cells at 7 days post SB treatment with the Dox-inducible SES lentiviruses to generate putative OECs in the presence of Dox. As shown in Supplementary Figures 2B and 2C, qRT-PCR and Western blot analyses indicated that both the SES genes and proteins were significantly overexpressed. Moreover, co-immunostaining for Sox2 and Six1 revealed that Sox2 and Six1 were co-expressed in single cells (Supplementary Figure 2D). Notably, the Sox2 staining indicated that the viral infection efficiency was about 43.7% (Supplementary Figures 2E,F). In the absence of Dox, no expression of the inducible SES was observed at either the mRNA or protein level, confirming the validity of the tunable SES overexpression.

**FIGURE 3:**
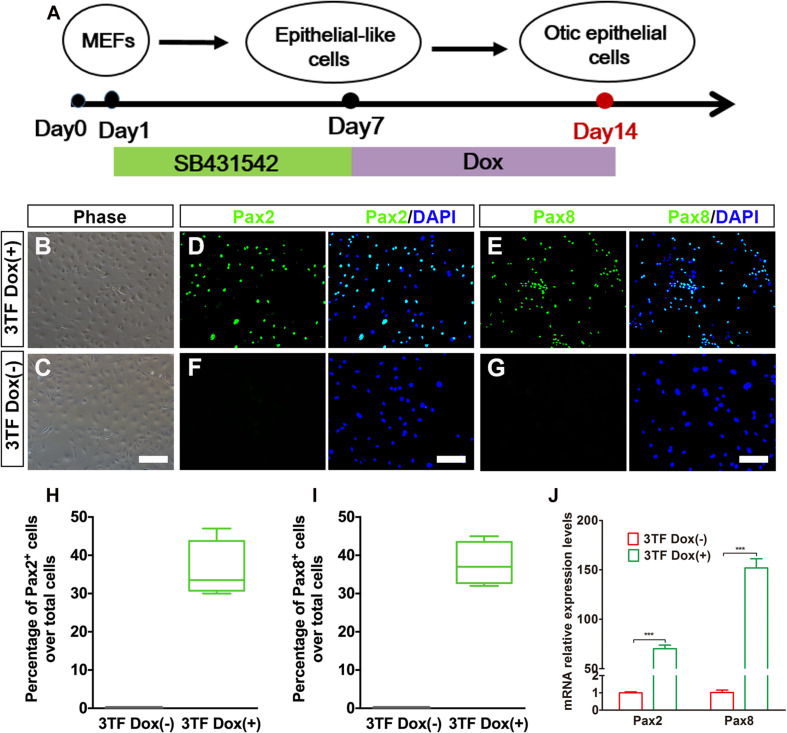
Inducible expression of Six1, Eya1, and Sox2 promotes the conversion of mouse embryonic fibroblast (MEF)-derived epithelial-like cells into otic epithelial cells (OECs). **(A)** MEF induction protocol including the sequential treatment with SB and doxycycline (Dox). **(B,C)** Morphology of OECs (OECs). **(D,E)** Immunostaining analysis of Pax2 and Pax8 expression in MEF-derived epithelial-like cells treated with Dox [3TF Dox (+)]. **(F,G)** Immunostaining analysis of Pax2 and Pax8 expression in MEF-derived epithelial-like cells treated without Dox [3TF Dox (-)]. **(H,I)** Frequencies of immunopositive cells for Pax2 **(H)** and Pax8 **(I)** in MEF-derived cells with or without 3TF Dox. Without Dox, immunopositivity for Pax2 and Pax8 was absent. In contrast, a remarkable number of immunopositive cells were detected in MEF-derived cells with Dox. Error bars represent the SEM. *n* = 4. **(J)** Quantitative real-time PCR analysis of the mRNA levels of OEC genes (Pax2 and Pax8) under different culture conditions. MEF-derived cells cultured without Dox were used as controls. Data were collected from at least three separate experiments and are shown as means ± SEM. Statistical significance was tested by Student’s *t*-test and expressed as **p < 0.01, ***p < 0.001 compared with the control (scale bar, 25 μm).

To evaluate whether OECs could be obtained from MEF-derived epithelial-like cells by overexpression of 3TF, we subjected the MEF-derived epithelial-like cells to a conversion protocol in which Dox treatment was initiated at day 7 and maintained during the following 7 days till day 14, when these cells were collected for analysis (Figure 3A). As expected, we found that the induced cells formed colonies at days 7–14 following Dox treatment. Consistent with what was described by Chen W. et al. (2012), the cells in the colonies with Dox treatment showed a flat phenotype with a large amount of cytoplasm and formed epithelioid islands (Figures 3B,C). To verify OEC induction, we used antibodies against Pax2 and Pax8 (Hans et al., 2004; Oshima et al., 2010), two markers for OECs, and quantified the numbers of Pax2^+^ cells and Pax8^+^ cells after 7 days of 3TF treatment with or without Dox (Figures 3D–G). As a result, cells without Dox treatment did not express Pax2 and Pax8. By contrast, in the 3TF treatment with Dox group, we observed a remarkable induction of Pax2^+^ cells (36% ± 3.8%) and Pax8^+^ cells (37.8% ± 2.8%) (Figures 3H,I). In addition, qRT-PCR analyses also confirmed their extremely high transcript levels, as compared with the group receiving 3TF treatment without Dox (Figure 3J). Remarkably, removal of any one or two factors of the 3 TFs reduced the efficiency of OEC induction (Supplementary Figure 3). To ascertain that the induced Pax8^+^ cells were indeed epithelial cells, we conducted Pax8 double staining with an epithelial marker E-cadherin. As expected, all of the Pax8^+^ cells were double labeled by the E-cadherin antibody (Supplementary Figure 4A). Altogether, these observations suggest that co-expression of SES induces a rapid conversion of MEF-derived epithelial cells into OECs.

**FIGURE 4:**
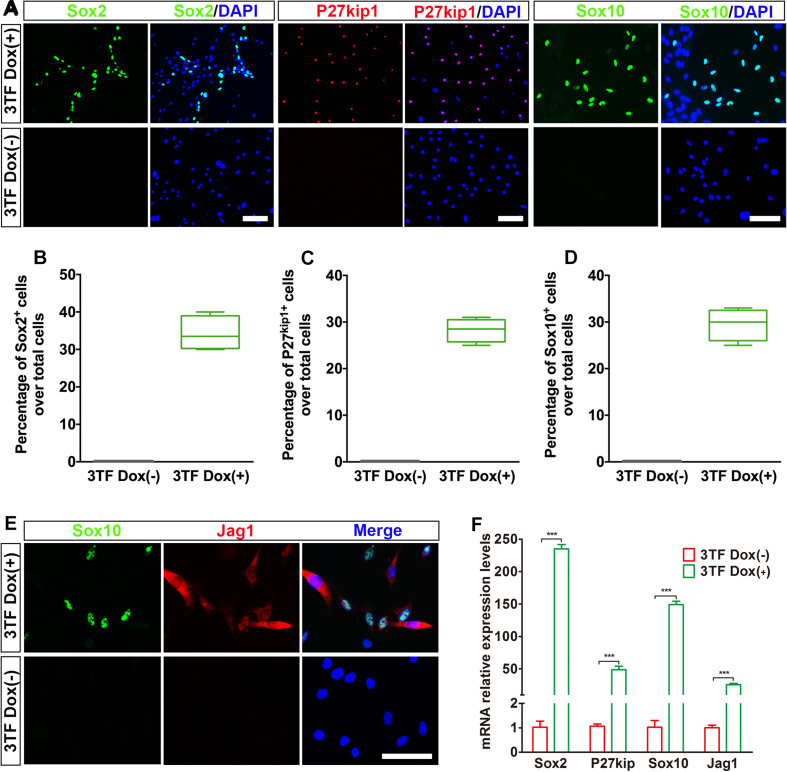
Otic epithelial cells (OECs) induced by Six1, Eya1, and Sox2 can display prosensory cell traits. **(A)** Immunostaining for Sox2, P27^kip1^, and Sox10 in mouse embryonic fibroblast (MEF)-derived OECs with or without doxycycline (Dox). Without Dox, immunopositivity for Sox2, P27^kip1^, and Sox10 was absent. In contrast, a remarkable number of immunopositive cells were detected in MEF-derived cells with Dox. **(B)** Frequencies of immunopositive cells for Sox2 in MEF-derived cells with or without Dox (*n* = 4). **(C)** Frequencies of immunopositive cells for P27^kip1^ in MEF-derived cells with or without Dox (*n* = 4). **(D)** Frequencies of immunopositive cells for Sox10 in MEF-derived cells with or without Dox (*n* = 5). **(E)** Double immunostaining of the prosensory cells using anti-Jag1 (red) and anti-Sox10 (green). **(F)** Quantitative real-time PCR analysis of the mRNA levels of prosensory cell genes (Sox2, P27^kip1^, Jag1, and Sox10) under different culture conditions. MEF-derived cells cultured without Dox were used as controls. Data were collected from at least three separate experiments and are shown as means ± SEM. Statistical significance was tested by Student’s *t*-test and expressed as **p < 0.01, ***p < 0.001 compared with the control (scale bar, 25 μm).

Previous studies suggested that during otic development, prior to HC production, there is a formation of the prosensory epithelium where HC progenitors and SCs are generated (Burns and Stone, 2017). To study whether the OECs induced from the MEFs via the above-mentioned protocol can form prosensory epithelial cells, we performed immunostaining for Sox2, P27^kip1^, Sox10, and Jag1, markers reminiscent of prosensory epithelial cells in the otic vesicle (Chen and Segil, 1999; Koehler et al., 2013; Wakaoka et al., 2013), in the cultures at days 16–18. Indeed, we observed a significant appearance of cells positive for these genes as compared with the control group (Figure 4A). The percentages of cells immunopositive for Sox2, P27^kip1^, and Sox10 in 3TF treatment with Dox cells were 34.3 ± 2.3% (Figure 4B), 28 ± 1.3% (Figure 4C), and 29.4 ± 1.5% (Figure 4D), respectively. Furthermore, Sox10^+^Jag1^+^ cells were also seen (Figure 4E). Control cultures not treated with Dox did not express Sox2, P27^kip1^, Sox10, and Jag1 (Figures 4A–E). Then these genes for prosensory-related markers were also analyzed using qRT-PCR. The data showed that mRNA expression levels of Sox2, P27^kip1^, Sox10, and Jag1 in cells with Dox were significantly higher than those in cells without Dox (*p* < 0.001), which was in agreement with the results of immunostaining (Figure 4F). In addition, to make sure that the induced Sox2-expressing cells represented epithelial cells, we performed Sox2 double staining with an epithelial marker E-cadherin. As expected, all of the Sox2-expressing cells were double labeled by the E-cadherin antibody (Supplementary Figure 4B). These results together imply that many of the OECs appear to differentiate further to become prosensory cell-like cells.

### Spontaneous Generation of Hair Cell-Like Cells Expressing Multiple Hair Cell Markers and Forming Cilia From Mouse Embryonic Fibroblast-Derived Otic Epithelial Cells

**FIGURE 5:**
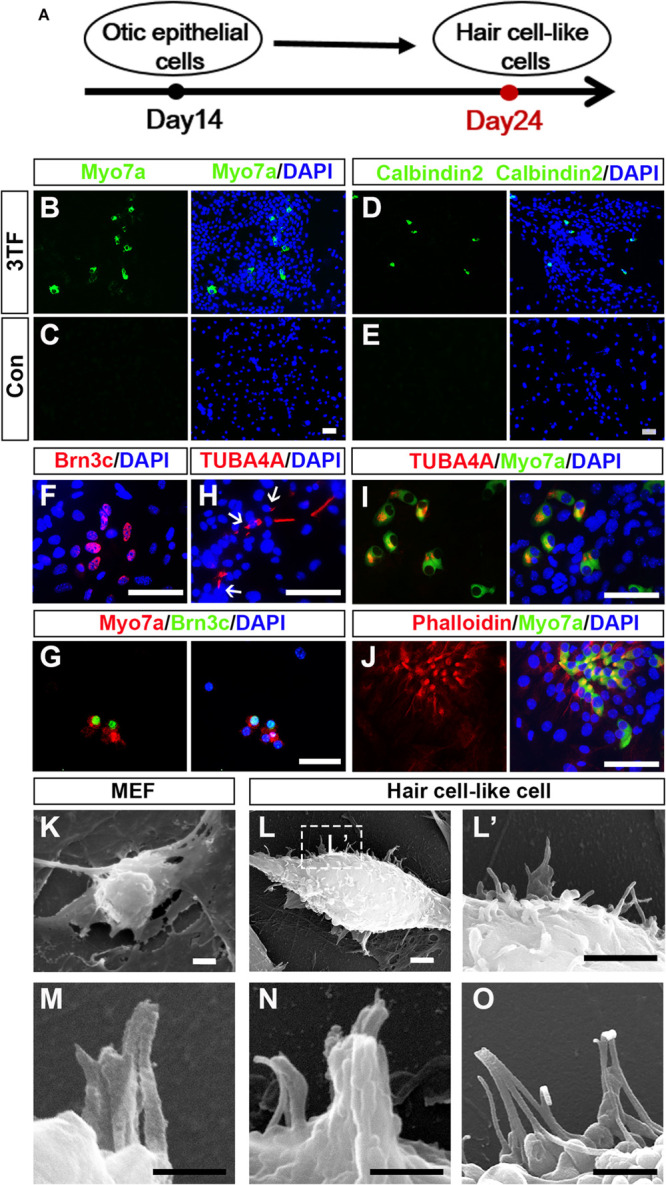
Mouse embryonic fibroblast (MEF)-derived otic epithelial cells (OECs) can give rise to hair cell-like cells (HCLs) exhibiting hair cell markers. **(A)** A schematic differentiation protocol from otic epithelial cells to HCLs. **(B,C)** Immunostaining for Myo7a in MEF-derived HCLs with (B) or without 3TF’s effect **(C)** at earlier stages. Strong upregulation of Myo7a is detected only in cells with 3TF’s effect at earlier stages. **(D,E)** Immunostaining for Calbindin2 in MEF-derived HCLs with **(D)** or without 3TF’s effect **(E)** at earlier stages. Strong upregulation of Calbindin2 is detected only in cells with 3TF’s effect at earlier stages. **(F)** Immunostaining analysis of Brn3c in HCLs with 3TF’s effect at earlier stages. **(G)** Double labeling for Myo7a (red) and Brn3c (green) in HCLs with 3TF’s effect at earlier stages. **(H)** The immunostaining with a kinocilium marker, TUBA4A antibody, in HCLs induced by the 3TF at earlier stages. **(I)** Double labeling for Myo7a (green) and TUBA4A (red) in HCLs induced by 3TF at earlier stages. **(J)** Double labeling for Myo7a (green) and Phalloidin (red) in HCLs induced by 3TF at an earlier stage. **(K)** The surface of the MEFs had no cilia. **(L)** Morphology of bundle-like structures protruding from the cell surface of an HCLs, as observed by SEM. **(L’)** The boxed regions at high magnification. **(M–O)** Noticeable inter-cilia links existed among the tips of ciliary structures in HCLs. Scale bars, 25 μm for **(B–J)**; 3 μm for **(K–L’)**; 500 nm for **(M,N)**; 1 μm for **(O)**.

As induction continues, we rendered OECs to a differentiation protocol in which Dox was removed after 7 days of treatment (Figure 5A). We then examined expression of markers for HC such as Myo7a, Calbindin2, or Brn3c to explore whether OECs could give rise to HCLs. Indeed, some of the OECs maintained in the culture for an additional 10 days became immunocytochemically positive for Myo7a or Calbindin2 (Figures 5B,D), two HC markers. On average, from 10^4^ initially plated cells, approximately 129 ± 39 cells were Myo7a^+^, and 105 ± 21 cells were Calbindin2^+^ (*n* = 4). In sharp contrast, no staining was observed in the cells without 3TF’s effect at earlier stages (Figures 5C,E). Furthermore, to confirm that the Myo7a^+^ cells are epithelial cells, we also carried out double staining of Myo7a^+^ cells with an epithelial marker E-cadherin. As expected, all of the Myo7a^+^ cells were double labeled by the E-cadherin antibody (Supplementary Figure 4C). In addition, we found that some cells were also positive for Brn3c (Figure 5F), that is, an HC marker and a TF required for HC survival and maturation (Xiang et al., 1998; Liu Q. et al., 2016). Per 10^4^ plated cells, about 153 ± 50 Brn3c^+^ cells were detected (*n* = 4). Notably, under this culture condition, from 10^4^ initially plated cells, approximately 18 ± 9 Brn3c^+^Myo7a^+^ cells (Figure 5G) were seen (*n* = 3).

Although these cells displayed many molecular characteristics specific of HCs, it was unclear if these cells had grown ciliary bundle-like structures. To address this issue, we performed immunostaining using an antibody against acetylated-α-Tubulin (TUBA4A), which was reported to label the kinocilium (Koehler et al., 2013, 2017), and indeed observed TUBA4A^+^ cilia in HCs (about 110 ± 27 in per 10^4^ plated cells, *n* = 4; Figure 5H). Moreover, these TUBA4A^+^ cells were also Myo7a^+^ cells (Figure 5I). Per 10^4^ plated cells, approximately 13 ± 8 TUBA4A^+^ Myo7a^+^ cells were detected (*n* = 3). Consistent with the above-described findings, we performed Phalloidin staining, which is often used to label the stereocilia of HCs (Koehler et al., 2017; McLean et al., 2017). We found that the induced HCLs showed expected Phalloidin staining with a concentration at the tip of the cell, which were also double labeled by Myo7a antibody (*n* = 3) (Figure 5J). In addition, we observed a small subset of Myo7a^+^ cells that formed a rudimentary apical bundle (Oshima et al., 2010; Chen W. et al., 2012; Costa et al., 2015), expressing Espin (Supplementary Figure 5). Furthermore, to provide additional evidence for the formation of the HC unique bundle structures, we examined the surface of the cells at 24 days of culture by scanning electron microscopy (SEM). In agreement with the immunostaining data, SEM revealed that elongated membrane protrusions reminiscent of ciliary bundles were present on the surface of some HCLs compared with control MEFs that did not display any of those structures (Figures 5K–L’). In addition, these putative bundles exhibited diverse arrangements. Importantly, the tips of ciliary bundle-like structures were also linked together (Figures 5M–O), an important feature related to stereocilary bundles (Costa et al., 2015). These results indicated differentiation of MEF-derived OECs to give rise to HCLs that display ciliary bundle-like structures.

As an attempt, we performed induction experiments by culturing the cells under three-dimensional (3D) conditions with a hope that a better HC morphology can be developed (McLean et al., 2017). As shown in Supplementary Figure 6, Myo7a immunostaining revealed that typical pear-shaped HCs were seen, which displayed a big cell body with a large nucleus at the bottom of the cell.

### The Hair Cell-Like Cells Generated in the Induction Cultures Acquire Mechanotransduction Channels

**FIGURE 6:**
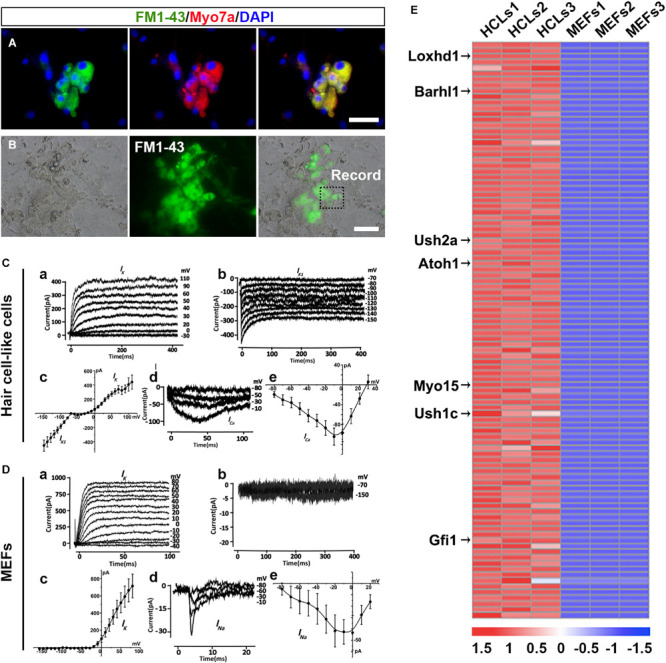
Ciliary structures and electrophysiological properties of hair cell-like cells (HCLs). **(A)** FM1-43^+^ cells co-expressing Myo7a. **(B)** White light image of a recording electrode on an FM1-43^+^ cell. **(C)** Electrophysiological properties of FM1-43^+^ cells. **(a)** The outward potassium channel currents were elicited by 10 voltage steps from the holding potential of –70 mV from mouse embryonic fibroblast (MEF)-derived HCLs (*n* = 10, Peak *I*_k__1_: 439.89 ± 98.52 pA at 110 mV). **(b)** The inward potassium channel currents were recorded from the holding potential of –70 mV by applying voltage in 10-mV decrements from MEF-derived HCLs (*n* = 10, Peak *I*_k__1_: –452.74 ± 78.28 pA at –150 mV). The inward and outward potassium channel currents resembled those recorded in pre-hearing mouse cochlear, with HCLs differentiated from human fetal auditory stem cells. **(c)** The average current–voltage (I–V) curve for outward and inward potassium current. **(d)** Example of Ca^2+^ current elicited from MEF-derived HCLs (*n* = 3, Peak Ica: –104.45 ± 17.18 pA at –10 mV). The hyperpolarized activation range of *I*_*Ca*_ indicated the presence of an L-type Ca^2+^ channel containing the Cav1.3 subunit, as previously reported in mammalian cochlear HCs. **(e)** The average current–voltage (I–V) curve of Ca^2+^. **(D)** Electrophysiological properties of MEFs. **(a)** The outward potassium channel currents were elicited from MEFs (*n* = 8, Peak *I*_k__1_: 714.97 ± 138.28 pA at 80 mV). **(b)** No obvious inward currents were recorded by applying voltage in 10-mV decrements (*n* = 8). **(c)** The average current–voltage (I–V) curve for outward and inward potassium current. **(d)** Small sodium current was elicited from MEFs in voltage injection in 10-mV increments (*n* = 3, Peak *I*_*Na*_: –43.32 ± 15.24 pA at 0 mV). **(e)** The average current–voltage (I–V) curve of sodium current. **(E)** Heat maps depicting the relative fold changes for the expression of HC-related genes between HCLs and MEFs for the top 100 genes (scale bars, 25 μm).

To explore whether HCLs expressed functional mechanotransduction channels, we added FM1-43 dye into the culture (Hu and Corwin, 2007). We found that FM1-43 rapidly entered into the Myo7a^+^ cells, indicating that these HCLs behaved like functional HCs (Figure 6A). Moreover, we studied whether the FM1-43^+^ cells respond to mechanical stimulation. We recorded membrane properties of the FM1-43^+^ cells by examining their voltage-dependent currents (Figure 6B). Nineteen FM1-43^+^ cells at day 24 were successfully recorded. Ten of these cells were positive for outward and inward K^+^ currents (IK and IK1) in the presence of KCl in the internal solution (Figures 6C-a–c), and three of these cells expressed inward Ca^2+^ current (*I*_*Ca*_) in the presence of CsCl in the internal solution (Figures 6C-d,e). The analysis of IK, IK1, and Ica suggests that the cells differentiated from OECs are the hair-cell-like cells (Chen W. et al., 2012). We also detected the electrophysiological profile of MEFs. Outward potassium current could be evoked while no IK1 current was elicited (Figures 6D-a-c).Voltage-gated sodium channel currents were detected with stimulation (Figures 6D-d,e), which could be blocked by 1 μM tetrodotoxin (TTX) perfusion. Therefore, the capability to take up FM1-43, the morphology of hair bundles, and the electrophysiological properties of HCLs suggested that MEF-derived OECs have differentiated into functional HCs.

### The Transcriptional Profiling of Hair Cell-Like Cells Exhibits a Selective Hair Cell Signature

To determine whether the transcriptional profiling of HCLs exhibits a selective HC signature, we carried out RNA-seq analysis of the HCLs at the end of induction compared with MEFs. The MEF-derived HCLs were incubated with FM1-43 dye (5 μM; Biotium) at room temperature for 10 s and rinsed with the culture medium. Then we obtained the FM1-43^+^ populations of HCLs with green fluorescence for transcriptional profiling by fluorescence-activated cell sorting (FACS) prior to RNA-seq experiments. We particularly focused on some genes known to be functionally relevant to inner ear HC development/function (Costa et al., 2015; Scheffer et al., 2015). Among the 32,007 detected genes in our samples, 15,433 genes showed differential expression. Out of 15,433 genes, 8,562 were upregulated (PADJ < 0.05). Among the 8,562 genes, more than 60 genes known to participate in the formation of HC were significantly upregulated. We then examined the positions of these genes within the ranked list. We found that more than half of them (34 HC-related genes) were positioned among the top 1,500 genes of this ranking (Supplementary Figure 7A and Supplementary Table 2). Moreover, 27 upregulated genes were in the top 1,000, and 14 were in the top 100. Transcripts from well-known HC-related genes such as Loxhd1, Barhl1, Ush2a, Atoh1, Myo15, Ush1c, and Gfi1 were in the top 100 genes most enriched in HCLs (Figure 6E). Importantly, as shown in Supplementary Figure 7B, qRT-PCR analysis validated the upregulation of the 14 HC-related genes by the HCLs (Costa et al., 2015; Scheffer et al., 2015). Consistently, the RNA-seq experiment also revealed a downregulation of several typical mesenchymal genes including Snail1, Snail2, Zeb2, and Vimentin (Supplementary Figure 8A), which were also verified by qRT-PCR analysis (Supplementary Figure 8B). Thus, the transcriptional profiling analysis of HCLs indicates a specific HC signature and further supports the notion that the induced cells are HCLs.

### Production Efficiency of Hair Cell-Like Cells Is Enhanced by Wnt Signaling Activation

**FIGURE 7:**
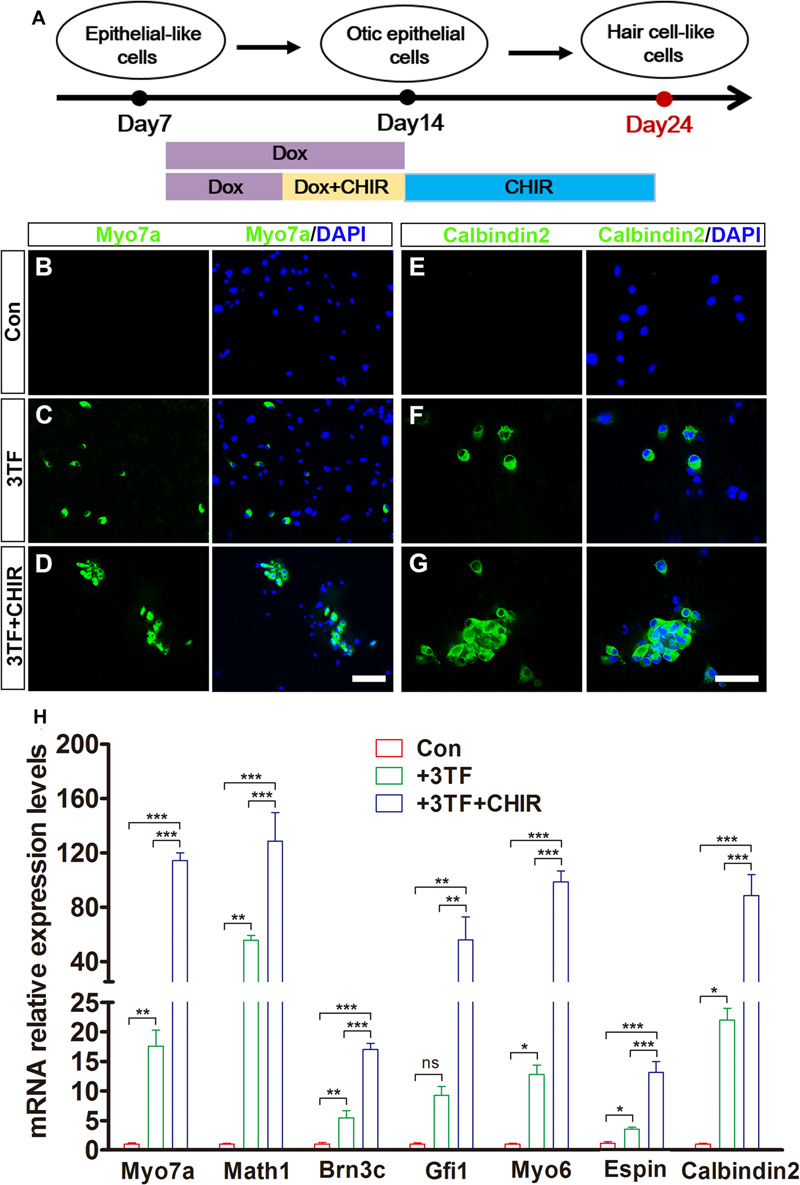
Production efficiency of hair cell-like cells (HCLs) is enhanced by Wnt signaling activation. **(A)** A schematic drawing of the induction procedure from epithelial-like cells (ELCs), including the sequential treatment with doxycycline (Dox) and CHIR. **(B–D)** Immunostaining analysis shows that Myo7a expression is higher in mouse embryonic fibroblast (MEF)-derived HCLs with 3TF + CHIR sequential treatment **(D)** than with 3TF treatment **(C)**. MEF-derived cells cultured without Dox were used as control **(B)**. **(E–G)** Immunostaining analysis shows that Calbindin2 expression is higher in MEF-derived HCLs with 3TF + CHIR sequential treatment **(G)** than with 3TF treatment **(F)**. MEF-derived cells cultured without Dox were used as control **(E)**. **(H)** Quantitative real-time PCR analysis of HC-specific gene expression under different induction conditions. Data are shown as mean ± SEM (*n* = 3). ns, not significant, *p < 0.5, **p < 0.01, ***p < 0.001 (scale bars, 25 μm).

Given that Wnt signaling activation is important for otic development and HC differentiation *in vivo* and enhances the formation of inner ear organoids *in vitro*, we next examined whether the efficiency of 3TF-induced HC production could be enhanced by addition of a Wnt signaling activator (Chai et al., 2012). We exposed 3TF-derived OECs to 4 days of Dox treatment in a combination with the CHIR99021(CHIR), a GSK3β inhibitor that activates the Wnt pathway (Liu X. P. et al., 2016; Koehler et al., 2017). Then, CHIR was kept continuously after Dox was withdrawn (Figure 7A). Remarkably, the numbers of cells immunopositive for Myo7a (Figures 7C,D) in 3TF + CHIR group was significantly higher than the 3TF only group (from 10^4^ initially plated cells, approximately 259 ± 33 cells vs. 125 ± 20 in 3TF group, *n* = 4, Figure 7H). Similar results were obtained by immunostaining for Calbindin2 (Figures 7F,G, from 10^4^ initially plated cells, approximately 206 ± 23 vs. 99 ± 18 cells, *n* = 4), another HC marker. No Myo7a or Calbindin2-expressing cells were seen in the control group (without 3TF and CHIR treatment, referred here as Con) (Figures 7B,E). In addition, mRNA expression levels of HC genes, including Myo7a, Atoh1, Brn3c, Gfi1, Myo6, Espin, and Calbindin2, were significantly elevated by CHIR treatment in the 3TF-induced OECs (Figure 7H), confirming that CHIR enhances HC production efficiency.

## Discussion

Hair cells are located in the inner ear sensory epithelium, which was originated from the otic placode during embryogenesis following a temporospatial program (Kelley, 2006). By mimicking and recapitulating the development of inner ear HCs *in vivo*, we designed our experiments to use a stepwise induction procedure to enable generation of functional HCs from MEFs. This stepwise method includes (1) MET of MEFs into epithelial-like cell; (2) conversion of MEF-derived epithelial-like cells into the OECs via inducible expression of Six1, Eya1, and Sox2; (3) differentiation of OECs into HCLs through an intermediate prosensory epithelial cell step; and (4) improving the production efficiency of HCLs by activating the Wnt pathway. We demonstrated successfully that as time proceeds; with the temporal order of induction, we observed stepwise expression of genes specific for the above-mentioned, related cell types at each of the steps. Therefore, the current study provides a novel strategy to generate inner ear HCs from MEFs.

In addition to verification of expression of HC-specific genes and proteins, the present study also confirmed formation of ciliary bundle-like structures on the surface of the induced HCLs via acetylated-α-Tubulin and Espin immunostaining and SEM. More importantly, our present work also demonstrated that the induced HCLs expressed functional mechanosensory channels as evidenced by the rapid infiltration of FM1-43 and acquisition of the electrophysiological features. Additional supports include a typical HC morphology in 3D induction cultures and realization of expression of more than 100 HC-selective genes based on the transcriptional profile analysis. Therefore, our stepwise strategy leads to a successful production of HCLs with immunocytochemical, morphological, electrophysiological, and transcriptional signature properties.

It is worth emphasizing that epithelial-to-mesenchymal transition (EMT) and MET are dynamic processes that have been shown to be important for embryogenesis (Chen J. et al., 2012; Liu et al., 2013). While EMT is involved in the formation of iPSCs from fibroblasts (Forte et al., 2017), MET is essential for differentiation of the stem cells into epithelial cell lineages (Li et al., 2010; Shu and Pei, 2014). The present study employed the concept of developmental biology to convert the MEFs into inner ear HCs, by initially switching to a different cell lineage, that is, epithelial cells. Our work is consistent with a previous study in a lower vertebrate system by Cowen and coworkers, reporting that while immortalization of the avian inner ear epithelial SCs can be achieved via an EMT process, differentiation of the immortalized SCs needs go through a MET path (Hu and Corwin, 2007). By addition of a TGF-β signaling inhibitor, SB431542, in the culture for 7 days, many of the MEFs became epithelial-like cells, based on their morphological changes and high expression level of E-cadherin, EpCAM, and ZO-1. The findings in current experiments are in agreement with others, reporting that MET initiates a reprogramming of mouse fibroblasts by suppressing TGF-β signaling (Li et al., 2010). In this way, the MET serves as a first step to recapitulate development process for the conversion of MEFs into inner ear HCs, which involves a switch from one cell lineage to another cell lineage.

Our results reinforce the notion that SES are core TFs of the genetic network participating in HC fate determination and differentiation. Sox2 is a TF that is required for specification of prosensory cells (Dabdoub et al., 2008). For instance, mutant mice that lack Sox2 in the inner ear show a loss of HCs, confirming the requirement for Sox2 in prosensory development (Kiernan et al., 2005). The transcription coactivator phosphatase Eya1 and its cofactor homeodomain protein Six1 are also necessary for HC development. Several studies have indicated inactivation of Eya1 or Six1 leads to an early arrest of otic development in mice (Xu et al., 1999; Ozaki et al., 2004). Moreover, Eya1/Six1 are co-expressed with Sox2 in the inner ear sensory progenitors. Both Atoh-dependent and Atoh-independent pathways are coordinately activated by Eya1/Six1, which in turn induce the expression of Brn3c so as to achieve differentiation of HCs (Ahmed et al., 2012). It is worth noting that even though Sox2 is necessary for the formation of prosensory domain, it also functions as an inhibitor by suppressing the effects of Atoh1 on induction of HC differentiation (Ahmed et al., 2012). To overcome this issue, we utilized a Tet-on gene expression system that enables Dox-inducible expression of SES. In this way, we were able to achieve induction of inner ear HCs that express multiple HC markers and acquire cilia and functions. Although overexpression of GFI1, Pou4f3, and ATOH1 together has been previously shown to induce conversion of human fibroblasts toward the HC lineage, that approach only allowed generation of cells expressing HC markers but failed to show their functions (Duran Alonso et al., 2018). In our experiments, following the reprogramming of MEFs to epithelial-like cells, transient overexpression of these three TFs in MEF-derived epithelial-like cells with Dox treatment at days 7–14 induced a lineage conversion of epithelial-like cells to OECs (Figure 3). As induction continues, OECs spontaneously differentiate into HCLs expressing multiple HC markers. Furthermore, these induced HCLs grow cilia and stereociliary bundle-like structures and acquired functional mechanosensory channels. Therefore, our Tet-on tunable gene expression system appears to be important for the maturation and acquisition of the functions of induced HCs.

It is also interesting to point out that activation of Wnt signaling pathway in the present experiments enhanced the HC induction rate, which is consistent with previous studies reporting the involvement of Wnt signaling in the otic progenitor cell (OPC) proliferation and differentiation (Chai et al., 2012). With the use of a Wnt pathway activator, CHIR99021, at the late stage when the OPCs are produced, the HC production rate is significantly enhanced. These results imply that activation of the Wnt signaling may be considered in future cell replacement therapy for hearing loss.

In conclusion, our study presents a new approach to produce functional inner ear HCs from somatic cells by using a novel stepwise protocol, providing a new working system for illumination of the developmental mechanisms of the HCs in the inner ears. More importantly, this approach might offer a cell replacement therapy for hearing loss in the future. To apply this strategy for the aim of HC regenerative in clinic, it is warranted to determine if human fibroblasts and other somatic cells can be successfully transdifferentiated into functional HCs.

## Materials and Methods

### Fibroblast Isolation and Cell Culture

Mouse embryonic fibroblasts were isolated from C57/BL6 mouse embryos at E13.5. All experiments that we performed were approved by the Ethics Committee at Shanghai Renji Hospital and followed standard established guidelines by Ministry of Health of China. Great attention was paid to remove the head, arms, legs, spinal cord, and internal organs of the embryos. The remaining tissue was dissociated manually in 0.25% trypsin (Sigma), kept at 37°C to obtain a single-cell suspension, and then plated in T75 flasks. MEFs were expanded to two passages or three passages (named as P2 or P3) in DMEM in which we supplemented with 10% FBS, as well as 1% penicillin (Life Technologies) at 37°C. The P2 or P3 MEFs were characterized by immunofluorescence to verify if they were pure without contamination of any HCs, SCs, or OECs using Vimentin, E-cadherin, Myo7a, Brn3c, P27^kip^, Sox10, Pax2, Pax8, Sox2, and Jag1. MEFs at P2 or P3 were used to perform the subsequent transdifferentiation experiments.

### Three-Dimensional Culture

Two days following transduction with 3TF, the cultured cells were incubated in TrypLE (Gibco) for 5 min at 37°C and collected in 15-ml tubes. After centrifugation for 5 min at 1,000 rpm, the dissociated cells were suspended in DMEM/F12 and strained using a 25-μm cell strainer to produce a single-cell suspension. The cells were then centrifuged again for 5 min at 1,000 rpm and re-suspended in 80:20 Matrigel (growth factor reduced, Corning): DMEM/F12. Then, we placed the Matrigel droplets (30–40 μl) containing the suspended cells in the center of each well of a 48-well plate, one droplet per well. To facilitate Matrigel rapid polymerization, the plate was incubated at 37°C for 15–20 min, after which the droplets were bathed in medium. Media were replaced every other day.

### Signaling Molecules and Recombinant Proteins

The following small compounds and recombinant proteins used in the study were as follows: human FGF2 (5–25 ng/ml; PeproTech), TGF-β inhibitor SB431542 (10 μM; Sigma), and GSK3β inhibitor CHIR99021 (3 μM; Sellect).

### RNA Preparation, PCR, and Real-Time Quantitative RT-PCR

Total RNAs were prepared from the cells using Zymo Research’s Quick-RNA MicroPrep Kit. cDNA was synthesized by reverse transcription with Takara’s the PrimeScript RT reagent Kit and oligo (dT) primers based on the manual. Real-time quantitative polymerase chain reaction (qRT-PCR) was performed using Toyobo’s SYBR Green method at Applied Biosystems’ Prism 7900 HT apparatus. Relative expression of mRNA was calculated by normalization of them to GAPDH mRNA. All the primer pairs were listed in Supplementary Table 1.

### Immunofluorescence Assay

We used 4% paraformaldehyde to fix cultured cells for 15 min and rinsed three times in phosphate-buffered saline (PBS) for 10 min each. They were infiltrated with 0.3% Triton X-100 for 10 min at room temperature and rinsed three times in PBS. To block non-specific bindings, the preparations were incubated with 10% normal donkey serum and 0.1% Tween 20 in PBS for 1 h. One percent normal donkey serum and 0.1% Tween 2 were added to the primary antibody solution for overnight at 4°C. The primary antibodies used included the following: rabbit anti-Vimentin (1:200, BD Biosciences), mouse anti-E-cadherin (1:200, BD Biosciences), mouse anti-ZO-1 (1:50, Santa Cruz), rabbit anti-Pax2 (1:400, Thermo Scientific), rat anti-Pax8 (1:400, Abcam), goat anti-Jag1 (1:100, Santa Cruz), mouse anti-Sox10 (1:100, eBiosciences), rabbit anti-Sox2 (1:200, Abcam), mouse anti-P27^kip1^ (1:100, Abcam), mouse anti-Brn3c (1:100, Abcam), rabbit anti-Myo7a (1:200, Proteus), mouse anti-acetylated-α-Tubulin (1:100, Sigma), mouse anti-Espin (1:50, Santa Cruz), rabbit anti-Calbindin2 (1:200, ProteinTech). After being washed three times with PBS for 10 min each, Alexa Fluor 488 (1:200, Life Technologies) or 594 (1:400, Life Technologies) conjugated, anti-rabbit, anti-goat, or anti-mouse secondary antibodies (Molecular Probes, Invitrogen) were used to detect primary antibodies. Nuclei were counterstained with 4′,6-diamidino-2-phenylindole (DAPI; 1:500, Sigma-Aldrich). After incubation for 1 h at room temperature and being washed three times with PBS, their fluorescence images were captured using inverted fluorescence microscopy or confocal laser scanning microscopy.

### Scanning Electron Microscopy

Undifferentiated MEFs and cells differentiated for 24 days were fixed in 2.5% glutaraldehyde that was made in 0.1 M of phosphate buffer (Sigma) overnight at 4°C. After three washes in PBS for 10 min each time and in 1% osmium tetroxide (Sigma) for 60 min each, the preparations were then dehydrated with a graded ethanol series and eventually processed with isoamyl acetate (Aladdin, Shanghai, China) for 20–30 min for critical point drying. Finally, they were examined using a scanning electron microscope (HitachiS-3000N, Japan), operated under a high vacuum at 5–10 kV at a working distance of 7–10 mm.

### FM1-43 Uptake Assay

To test whether the MEF-derived HCLs acquired functional mechanotransduction channels, the cultures were incubated with FM1-43 dye (5 μM; Biotium) at room temperature for 10 s and rinsed with the culture medium. The preparations were then fixed in 4% paraformaldehyde, counterstained with DAPI, and finally examined under a fluorescence microscope. Moreover, immunofluorescence for Myo7a was processed after FM1-43 dye staining to verify the identity of HCs. Fields were chosen randomly (*n* = 15).

### Electrophysiological Recordings

Whole-cell patch-clamp technique was used to record the membrane currents of cells differentiated for 24 days with an amplifier (EPC 10; HEKA Electronik). PatchMaster software (HEKA) at a Dell computer was used to filter the data at 1–3 kHz and to sample at 3–10 kHz. A vertical pipette puller (PC-10; Narishige) was used to pull patch pipettes from borosilicate capillary glass. The resistance of the electrode was 4–7 MΩ. For *I*_k__1_, *I*_k_, and *I*_*Na*_ acquirement, the patch electrodes were filled with 131 mM of KCl, 3 mM of MgCl_2_, 1 mM of EGTA-KOH, 5 mM of Na_2_ATP, 5 mM of HEPES, and 10 mM of sodium phosphocreatine (pH 7.3). For *I*_*Ca*_ recording, pipette was filled with recording solution (pH 7.3) containing 3 mM of MgCl_2_, 131 mM of CsCl, 1 mM of EGTA-KOH, 5 mM of Na_2_ATP, 1 μM of TTX, 5 mM of HEPES, and 10 mM of sodium phosphocreatine. Recording solution contained (in mM) 135 NaCl, 5.8 KCl, 1.3 CaCl_2_, 0.9 MgCl_2_, 0.7 NaH_2_PO_4_, 5.6 D-glucose, 10 HEPES, and 2 sodium pyruvate. We performed all recordings with the pH adjusted to 7.3–7.5 at room temperature (20–25°C). Cells used for electrophysiology assay were kept in DMEM/F12 containing FM1-43 for 10 s. The cells labeled with FM1-43 showed green fluorescence in the cytoplasm, which were then used for electrophysiological recordings. The cells were bathed in the external solution and visualized at an inverted phase-contrast microscope (TE-2000U; Nikon). Data were stored on a DELL computer for off-line analysis using Clamp fit and Origin software (Origin Lab). We considered the peak current as the maximum current when it appeared during the step depolarization. Membrane currents were elicited by applying voltage in 10-mV increments or decrements to the holding potential of −70 mV.

### RNA Sequencing Analysis

RNA from MEFs and HCLs induced for 24 days was extracted using the TRIzol reagent (Invitrogen). The RNA-seq work was performed by Novogene (Beijing, China). Sequencing libraries were produced using NEBNext^®^ UltraTM RNA Library Prep Kit for Illumina^®^ (NEB, United States) following the manufacturer’s manual, and index codes were added to assign sequences to each sample. Briefly, mRNA was purified by poly-T oligo-attached magnetic beads. The clustering of the index-coded samples was performed on a cBot Cluster Generation System using TruSeq PE Cluster Kit v3-cBot-HS (Illumina) according to the manufacturer’s instructions. As for data analysis, differential expression analysis of two groups was conducted using the DESeq2 R package (LABEL:GrindEQ__1_16_1_). The resulting p-values were adjusted using the Benjamini and Hochberg approach to control the false discovery rate. Genes with an adjusted *p*-value < 0.05 found by DESeq2 were regarded as differentially expressed. For each group, three biological replicates were used for the analysis. The data presented in the study are deposited in the (SRA) repository, accession number (PRJNA713364).

### Statistical Analysis

All experiments were performed independently with at least three biological replicates. Statistical analysis was performed using the GraphPad Prism software (version 5.0). Data were presented as mean ± SEM, and statistical significance was performed using Student’s *t*-test and expressed as *p* < 0.05 (^∗^), *p* < 0.01 (^∗∗^), and *p* < 0.001 (^∗∗∗^). The fraction of immunopositive cells among total cells was determined in a double-blinded fashion by observations of approximately 300 cells in each of 10 randomly selected microscopic fields per experiment.

## Data Availability Statement

The data presented in the study are deposited in the (SRA) repository, accession number (PRJNA713364).

## Ethics Statement

The animal study was reviewed and approved by Shanghai Renji Hospital and followed standard established guidelines by Ministry of Health of China.

## Author Contributions

W-QG conceived the concept. QY, HS, and YQ performed the experimental work. QY, HS, YQ, HX, H-BS, and W-QG contributed to the experimental design and data analysis. YQ, WL, LW, YW, ZJ, and S-KY supervised the study. QY, H-BS, and W-QG wrote the manuscript. All authors interpreted the data, discussed the results and approved the final version of the manuscript.

## Conflict of Interest

The authors declare that the research was conducted in the absence of any commercial or financial relationships that could be construed as a potential conflict of interest.

## Abbreviations

ESCs: embryonic stem cells
HCLs: hair cell-like cells
HCs: hair cells
iPSCs: induced pluripotent stem cells
MEFs: mouse embryonic fibroblasts
MET: mesenchymal-to-epithelial transition
OECs: otic epithelial cells
OPCs: otic progenitor cells
SCs: supporting cells
SES: Sox2/Eya1/Six1
TGF- β: transforming growth factor beta.
