# Supplementary material for: Stepwise Induction of Inner Ear Hair Cells From Mouse Embryonic Fibroblasts via Mesenchymal- to-Epithelial Transition and Formation of Otic Epithelial Cells
Source: Front Cell Dev Biol. 2021 Jun 17;9:672406. doi: 10.3389/fcell.2021.672406 (PMC8248816; doi:10.3389/fcell.2021.672406)
Supplement: Supplementary file 1 [file Data_Sheet_1.pdf]

Supplementary Figure and Figure Legends

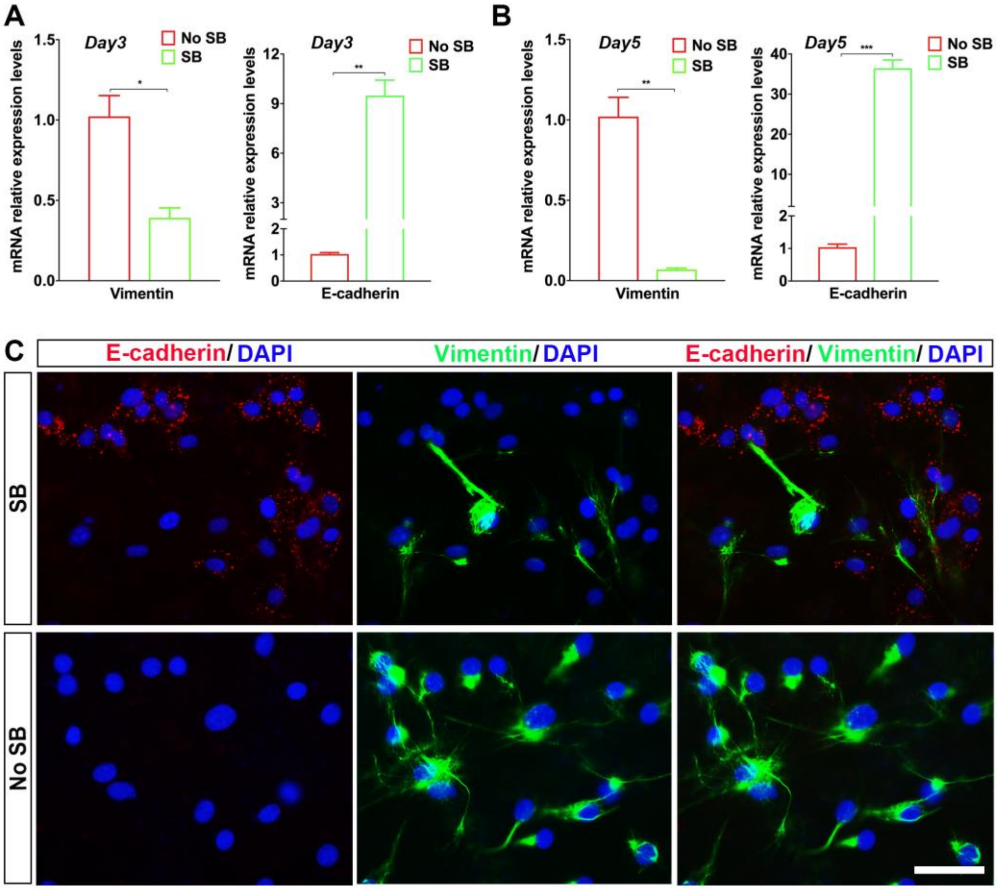

Supplementary Figure 1. qRT-PCR and immunostaining analysis of E-cadherin and Vimentin expression in the culture in the presence or absence of SB. (A) Quantitative RT-PCR analysis of mRNA levels of Vimentin and E-cadherin at the induction of day 3. MEFs in normal cultures were used as a control group, No SB. **(B)** Quantitative RT-PCR analysis of mRNA levels of Vimentin and E-cadherin at the induction of day 5. Data were collected from at least three separate experiments and are shown as means ± SEM. Statistical significance was analyzed using the Student's test; *p<0.05, **p<0.01, ***p<0.001. **(C)** Double immunostaining of Vimentin and E-cadherin of MEFs with or without SB treatment at the induction of day 5. (Scale bar, 25μm.)

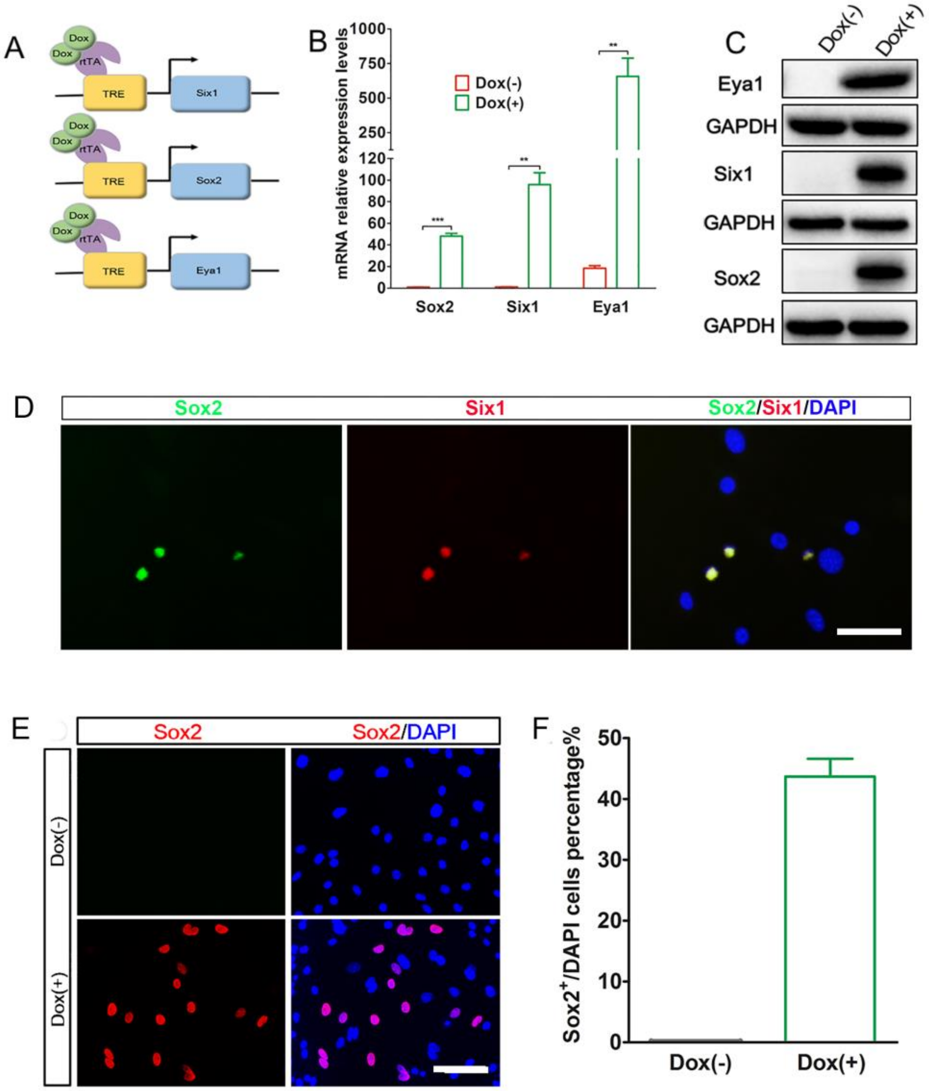

Supplementary Figure 2. Overexpression of three transcription factors in MEFs. (A) The Dox-inducible Six1, Eya1 and Sox2 lines used in this study. TRE, tetracycline responsive element; rtTA, reverse tetracycline trans-activator. **(B)** Significantly increased mRNA expression of the genes was observed compared to non-induced control cultures by qPCR analyses. Data were collected from at least three separate experiments and are shown as means ± SEM. Statistical significance was analyzed using the Student's test; **p<0.01, ***p<0.001. **(C)** Immunoblotting analysis verifies the overexpression of Eya1, Six1, Sox2 in lentiviral-transfected MEFs treated with Dox. **(D)** Double immunostaining of Sox2 and Six1 in lentiviral-transfected MEFs treated with Dox. **(E)** Immunofluorescent staining indicates that Sox2 were overexpressed in lentiviral-transfected MEFs treated without or with Dox. **(F)** The infection efficiency was presented as the percentage of $Sox2^+$ cells versus $DAPI^+$ cells. n≧3 experiments. Five to six representative visual fields for each of group were counted. (Scale bars, 25μm.)

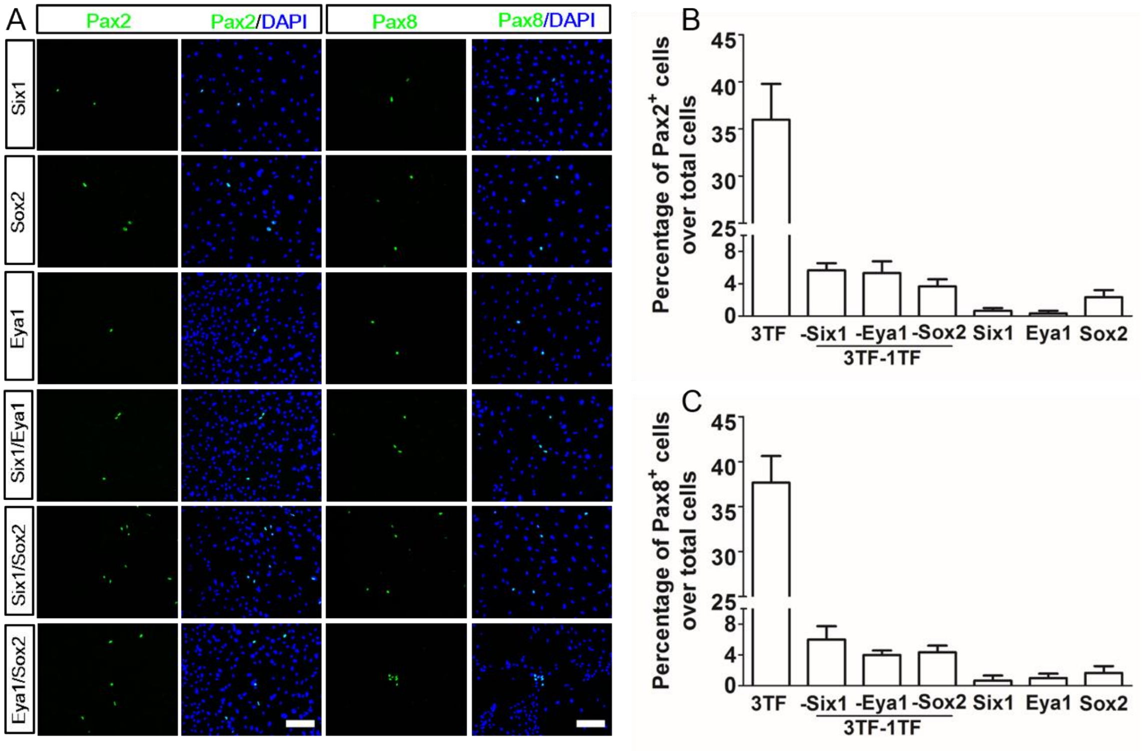

Supplementary Figure 3. The efficiency of OEC induction is relatively low in the absence of Six1, Eya1 or Sox2. (A) Immunostaining analysis of Pax2 and Pax8 expression in one factor or two factor induction cells. **(B)** Effects of individual or two factors withdrawal from 3TF on Pax2 positive cells formation. **(C)** Effects of individual or two factors withdrawal from 3TF on Pax8 positive cells formation. Error bars represent the SEM. (n=3 experiments, randomly selected 20x visual fields from triplicate samples). (Scale bars, 30μm.)

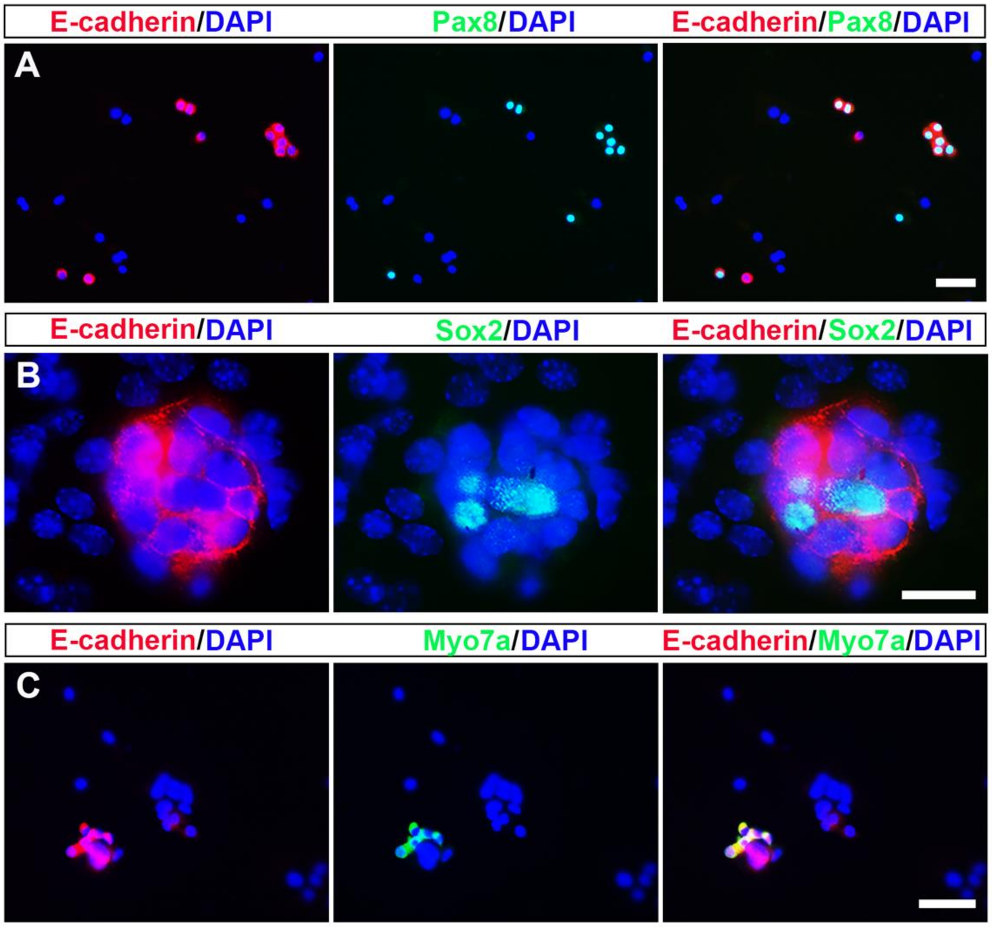

Supplementary Figure 4. The induced OECs and HCLs are epithelial cells. (A) Double immunostaining of Pax8 and E-cadherin for OECs. (B) Double immunostaining of Sox2 and E-cadherin for prosensory cells. (C) Double immunostaining of Myo7a and E-cadherin for HCLs. (Scale bars, 25μm for A and C; 15μm for B.)

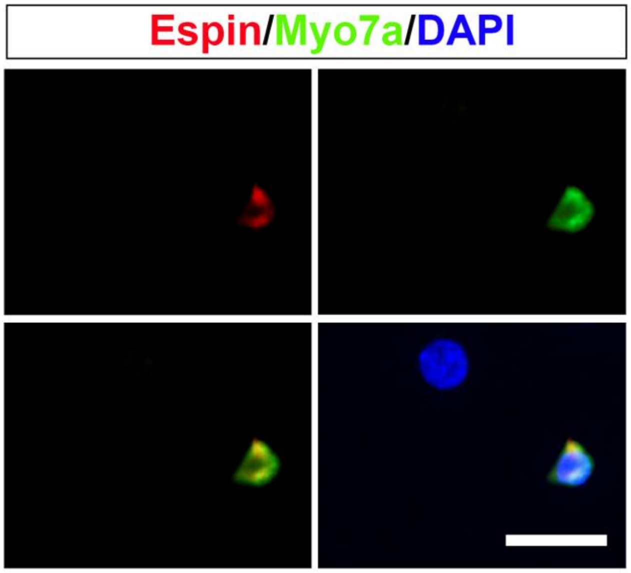

Supplementary Figure 5. Double immunostaining for Myo7a and Espin of HCLs.

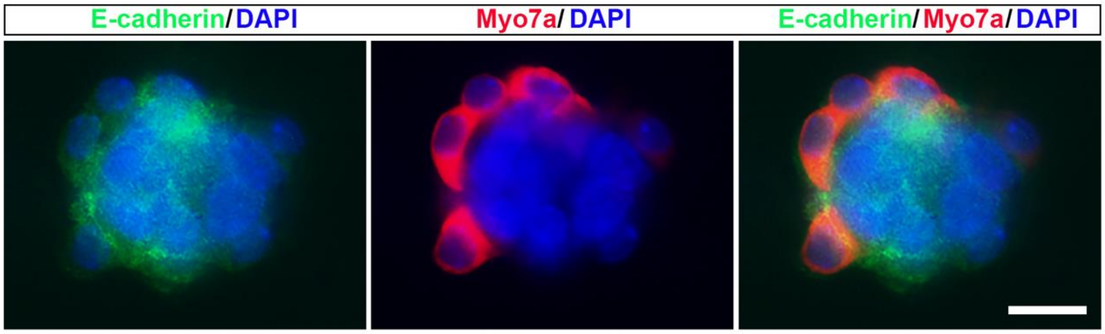

Supplementary Figure 6. Double immunostaining for Myo7a and E-cadherin of HCLs in 3D culture.

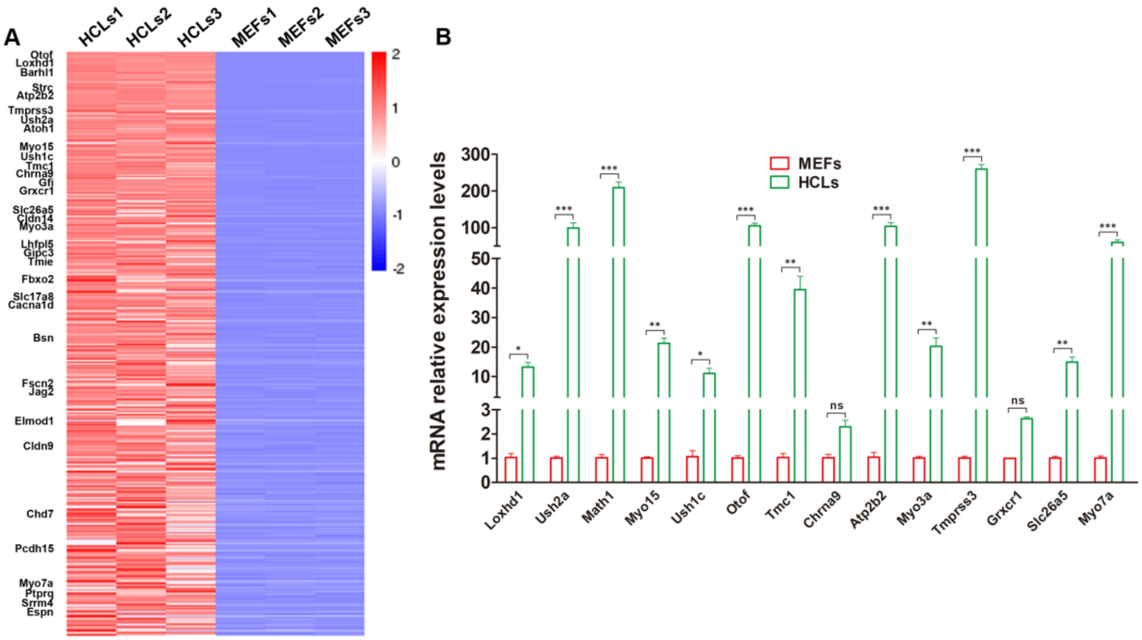

Supplementary Figure 7. Heat maps and qRT-PCR analysis of the HC-related genes expression between HCLs and MEFs. (A) Heat map depicting the relative fold changes for the expression of genes between HCLs and MEFs for the top 1500 upregulated genes, in which 34 HC-related genes were found. (B) qRT-PCR analysis of 14 HC-related genes expression between HCLs and MEFs for the top 1500 upregulated genes. Data are shown as mean ± SEM (n=3). ns, not significant, *p<0.5, **p<0.01, ***p<0.001.

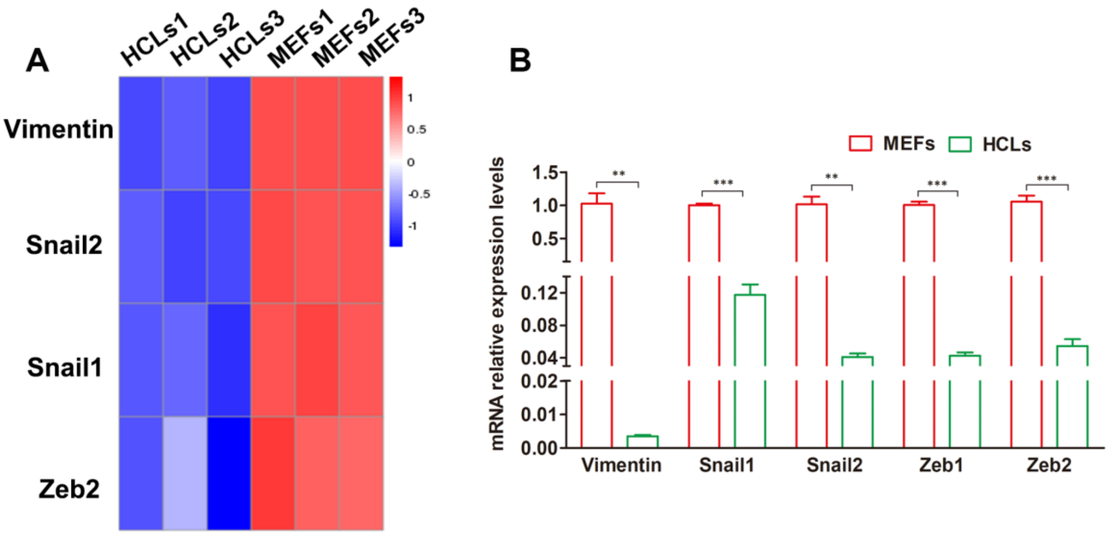

Supplementary Figure 8. Heat maps and qRT-PCR analysis of the mesenchymal related gene expression between HCLs and MEFs. (A) Heat map depicting the downregulation of several typical mesenchymal markers including Snail1, Snail2, Zeb2 and Vimentin (B) qRT-PCR analysis of these mesenchymal gene expression between HCLs and MEFs. Data are shown as mean ± SEM (n=3). *indicates p<0.5, ** marks p<0.01, ***indicates p<0.001.

Supplementary Table S1. Primers used in this paper

**Primers**		
**name**	**Primer1(5' to 3')**	**Primer2(3' to 5')**
E-cadherin	CAGGTCTCCTCATGGCTTTGC	CTTCCGAAAAGAAGGCTGTCC
EpCAM	GCGGCTCAGAGAGACTGTG	CCAAGCATTTAGACGCCAGTTT
ZO-1	ACCACCAACCCGAGAAGAC	CAGGAGTCATGGACGCACA
Pax2	AAG CCC GGA GTG ATT GGT G	CAG GCG AAC ATA GTC GGG TT
Pax8	ATGCCTCACAACTCGATCAGA	ACAATGCGTTGACGTACAACTT
Sox2	GCGGAGTGGAAACTTTTGTCC	CGGGAAGCGTGTACTTATCCTT
$P27^{kip1}$	TCAAACGTGAGAGTGTCTAACG	CCGGGCCGAAGAGATTTCTG
Sox10	ACACCTTGGGACACGGTTTTC	TAGGTCTTGTTCCTCGGCCAT
Jag1	CCTCGGGTCAGTTTGAGCTG	CCTTGAGGCACACTTTGAAGTA
Myo7a	AATCACATCAGGTACAGCGAAGA	CGGGGAAGTAGACCTTGTGGA
Atoh1	ATGCACGGGCTGAACCA	TCGTTGTTGAAGGACGGGATA
Brn3c	ATGCGCCGAGTTTGTCTCC	GGGCTTGAACGGATGATTCTTG
Gfi1	AGGAACGCAGCTTTGACTGT	TGAGATCCACCTTCCTCTGG
Myo6	GAGAGGCGGATGAAACTTGAGA	CTTCGGAGTGCCATGTCACC
Espin	CCACAGGCTACCTCTCTTGC	AGCAGCCACTTCACCACATC
Calbindin2	AGTACACCCAGACCATACTACG	GGCCAAGGACATGACACTCTT
Six1	ATGCTGCCGTCGTTTGGTT	CCTTGAGCACGCTCTCGTT
Eya1	TAACAGCTCGCCGTATCCAG	GTCCCAGATGAACACTCTCTCA
GAPDH	AACACAGTCCATGCC	TCCACCACCCTGTTGCTG

Supplementary Table S2. Selective HC-associated genes in the top 1500 genes

Gene name	log2 Fold increase	Rank（log2 Fold increase）
Otof	14.65988157	1/8562
Loxhd1	14.50839983	3/8562
Barhl1	13.49628412	9/8562
Strc	13.06369867	16/8562
Atp2b2	12.80905965	19/8562
Tmprss3	12.33919589	29/8562
Ush2a	12.11467274	35/8562
Atoh1	11.9528662	39/8562
Myo15	11.28602661	60/8562
Ush1c	11.20050799	65/8562
Tmc1	11.09131813	71/8562
Chrna9	10.98548503	76/8562
Gfi	10.76974469	86/8562
Grxcr1	10.53309992	95/8562
Slc26a5	10.29475826	113/8562
Cldn14	9.962923717	139/8562
Myo3a	9.896880331	147/8562
Lhfpl5	9.442237199	191/8562
Gipc3	9.278799123	203/8562
Tmie	9.016562771	239/8562
Fbxo2	8.577785469	306/8562
Slc17a8	8.002329792	437/8562
Cacna1d	7.927130086	464/8562
Bsn	7.419264147	638/8562
Fscn2	6.843442715	844/8562
Jag2	6.799423809	866/8562
Elmod1	6.693068488	907/8562
Cldn9	6.382732803	1073/8562
Chd7	6.13564595	1238/8562
Pcdh15	5.929606021	1380/8562
Myo7a	5.887364208	1414/8562
Ptprq	5.852568157	1436/8562
Srrm4	5.842876796	1445/8562
Espn	5.806995431	1469/8562
